# TPX2 is a novel prognostic marker for the growth and metastasis of colon cancer

**DOI:** 10.1186/1479-5876-11-313

**Published:** 2013-12-17

**Authors:** Ping Wei, Nu Zhang, Ye Xu, Xinxiang Li, Debing Shi, Yuwei Wang, Dawei Li, Sanjun Cai

**Affiliations:** 1 Department of Pathology, Fudan University Shanghai Cancer Center, Shanghai 200032, China; 2 Department of Colorectal Surgery, Fudan University Shanghai Cancer Center, Shanghai 200032, China; 3 Cancer Institute, Fudan University Shanghai Cancer Center, Shanghai 200032, China; 4 Department of Oncology, Shanghai Medical College, Fudan University, Shanghai 200032, China; 5 Department of Neurosurgery, 1st affiliated hospital of Sun Yat-sen University, Guangzhou 510080, China

**Keywords:** TPX2, Colon cancer, Tumorigenesis, Metastasis

## Abstract

**Background:**

We have previously demonstrated an aberrant overexpression of the microtubule-associated protein TPX2 in colon cancer using a genome-wide gene expression profiling analysis. Here, we aim to investigate its expression pattern, clinical significance, and biological function in colon cancer.

**Methods:**

TPX2 expression was analyzed in human colon cancer cell lines and tumor samples. The effect of TPX2 on cell proliferation, tumorigenesis, and metastasis was examined *in vitro* and *in vivo*.

**Results:**

TPX2 was overexpressed in 129 of the 203 (60.8%) colon cancer metastatic lesions, with the expression being significantly higher than that in primary cancerous tissue and normal colon mucosa. Overexpression of TPX2 was significantly associated with clinical staging, vessel invasion, and metastasis. In survival analyses, patients with TPX2 overexpression had worse overall survival and metastasis free survival, suggesting that deregulation of TPX2 may contribute to the metastasis of colon cancer. Consistent with this, suppression of TPX2 expression inhibited proliferation and tumorigenicity of colon cancer cells both *in vitro* and *in vivo*. Strikingly, we found that TPX2 knockdown significantly attenuated the migration and invasion ability of colon cancer cells, which was further shown to be mechanistically associated with AKT-mediated MMP2 activity.

**Conclusions:**

These findings suggest that TPX2 plays an important role in promoting tumorigenesis and metastasis of human colon cancer, and may represent a novel prognostic biomarker and therapeutic target for the disease.

## Background

Colorectal cancer is one of the leading causes of cancer-related deaths worldwide [1]. Approximately 50–60% of patients diagnosed with colorectal cancer develop colorectal metastases, and 80–90% of these patients have unresectable metastatic live disease [2-5]. However, the precise genetic changes responsible for the initiation and progression of colon cancer remain poorly understood. Therefore, there is a need to identify new gene targets and develop novel target-specific therapies.

TPX2, a microtubule-associated protein, is encoded by a gene located on human chromosome band 20q11.1 [6,7]. It is required for microtubule formation at kinetochores in mammalian cells, which is mediated through binding of the COOH-terminal domain of Xenopus kinesin-like protein 2 (Xklp2) to microtubules [8,9]. TPX2 is downstream of Ran-GTP and plays a central role in spindle formation. In the early stages of mitosis, TPX2 is released in a RanGTP-dependent manner [10,11], and interacts with Aurora A kinase. This results in the localization of Aurora A to the microtubules of the mitotic spindle, which then initiates spindle assembly [12]. The N-terminal domain of TPX2 interacts with Aurora A, thus protecting Thr288 in the T-loop of the kinase from dephosphorylation by Phosphatase Protein 1 [13]. Cells deficient in the Aurora A/TPX2 complex present short spindles, which results in mitotic failure [14]. TPX2 expression is tightly regulated during the stages of cell cycle, becoming detectable at the G1-S transit and disappearing at the completion of cytokinesis [15]. Therefore, TPX2 expression might provide a more precise evaluation of the proliferative behavior of tumor cells.

Recently, several tumors have been found to show aberrant expression of TPX2, such as copy number-driven overexpression from the amplicon on 20q11.2 in non-small-cell lung cancer [16], high mRNA and protein levels in pancreatic ductal adenocarcinomas [17], and in more than 50% of patients of giant-cell tumor of the bone [18]. However, no attempt has been made to investigate the expression of TPX2 in human colon cancer. In this study, we investigate the expression of TPX2 at the mRNA and protein level in human colon cancer, clarify the correlation between the TPX2 expression and clinicopathological parameters, and predict the underlying mechanism of its potential role in the proliferation and metastasis of colon cancer cells.

## Material and methods

### Patient information and tissue specimens

This study was approved by the Institutional Research Ethics Committee and written consents were obtained from all 203 patients with pathologically and clinically confirmed colon cancer. None of the patients had received radiotherapy or chemotherapy before surgery. Staging was based on pathological findings according to the American Joint Committee on Cancer (AJCC). Based on the tumor (T), node (N), and metastasis (M) classification system, we identified 24 cases at stage I, 81 at stage II, 80 at stage III, and 18 at stage IV. The matching adjacent noncancerous tissue, primary colon cancer tissue, and lymph node metastasis lesions from the 203 patients was fixed in formalin and embedded in paraffin for histological analysis and immunohistochemical studies. Fresh samples were dissected manually to remove connective tissues and were immediately stored in liquid nitrogen until western blot analysis.

### TMA construction and immunohistochemistry

The tissue array construction procedure has been described previously [19]. Sections (4-μm thick) of TMA slides were prepared and processed for immunostaining. The paraffin sections were de-paraffinized in xylene and rehydrated in a graded alcohol series, boiled with 10 mmol/L of citrate buffer (pH 6) for 10 min, and treated with 0.3% H_2_O_2_ for 10 min. The steps were performed using the Envision two-step method. The Envision and DAB Color Kit was purchased from Gene Tech Company Limited (Shanghai). The TPX2 anti-human rabbit polyclonal antibody was used at a dilution of 1:200, PBS was used as a negative control. Immunoreactivity was evaluated independently by two researchers in a blinded fashion. The evaluation was based on the staining intensity and extent of staining. The staining intensity was graded as follows, 0: no staining, 1+: mild staining, 2+: moderate staining, and 3+: intense staining. The staining area was scored using the following scale, 0: no staining of cells, 1+: <10% of tissue stained positive, 2+: 10–50% stained positive, and 3+: >50% stained positive. The sum of staining score (intensity + extension) index was designated as follows, 0–2: negative expression, 3–4: weak expression, and 5–6: strong expression.

### RNA extraction, reverse transcription (RT), and quantitative real-time PCR (qPCR)

RNA was isolated according to the manufacturer’s instructions (TRIzol, Invitrogen, USA). One microgram of total RNA from each sample was subjected to first-strand cDNA synthesis according to the manufacturer’s recommendations (Promega, USA). Quantitative PCR was performed on a Mastercycler® ep*realplex* (Eppendorf) with an IQTM SYBR Green Supermix Kit (BIO-RAD) according to the manufacturer’s protocol. TPX2 was amplified with the following primers: 5′-AGGGGCCCTTTGAACTCTTA-3′ (forward primer) and 5′-TGCTCTAAACAAGCCCCATT-3′ (reverse primer). GAPDH was used as an endogenous control with the following primers: 5′-CGGATTTGGTCGTATTGG-3′ (forward primer) and 5′-TCCTGGAAGATGGTGATG-3′ (reverse primer). The cycling conditions for TPX2 and GAPDH were as follows: one cycle at 95°C for 3 min; 40 cycles of 95°C for 15 s, and 60°C for 60 s. The specificity of the PCR amplification was validated by the presence of a single peak in the melting curve analyses. Each RT-qPCR experiment was repeated three times.

### Plasmids

For depletion of TPX2, a human siRNA sequence was cloned into the pSilencer™ 2.1-U6 puro Vector (Ambion) according to manufacturer’s protocol. The target sequence was 5′-AAGAATGGAACTGGAGGGCTT-3′. A scrambled siRNA (5′-GTACCGCACGTCATTCGTATC -3′) with no homology to the mammalian mRNA sequences was used as a negative control. Transfection of TPX2-shRNA or control-shRNA plasmid was performed using the Lipofectamine 2000 reagent (Invitrogen) according to the manufacturer’s instructions.

### 3-(4, 5-Dimethyl-2-thiazolyl)-2, 5-diphenyl-2H-tetrazolium bromide (MTT) assay

Cells were seeded in 96-well plates at an initial density of 0.2 × 10^4^ cells/well. At each time point, cells were stained with 100 μL sterile MTT dye (0.5 mg/mL, Sigma) for 4 h at 37°C, followed by removal of the culture medium and addition of 150 μL of dimethyl sulphoxide (DMSO) (Sigma, St. Louis, MO, USA). The absorbance was measured at 570 nm, with 655 nm as the reference wavelength. All experiments were performed in triplicate.

### Cell migration and invasion assays

Cell migration and invasion assays were conducted using a modified 24-well Boyden chamber with a membrane that was uncoated, or coated with Matrigel (BD Biosciences). Briefly, 24 h after transfection of both HCT116 and SW620 cells either with a control (mock or control shRNA) or TPX2 shRNA, the cells were harvested and resuspended in DMEM at a concentration of 5 × 10^4^ cells/mL. Cells prepared in 500 uL of DMEM were loaded in the upper wells, and a medium containing 20% FBS was placed in the lower wells as a chemoattractant stimulus. Cells that had migrated to the bottom surface of the filter were fixed, stained with H&E, and counted under a microscope in three randomly selected fields at a magnification of 200 × .

### Gelatin zymography assay

SW620 cells (2 × 10^5^ cells) were seeded in six-well plates and incubated overnight at 37°C. The cells were washed twice with Hanks’ balanced salt solution and cultured for an additional 24 h in serum-free medium. Culture supernatants were collected for collagenase activity assays. Culture supernatants (40 μL) were resolved on a 7.5% sodium dodecyl sulfate polyacrylamide gel that contained 1 mg/mL gelatin (Sigma Chemical Co., St. Louis, MO). The gel was washed for 30 min at room temperature in wash buffer (50.0 mM Tris–HCl [pH 7.5], 15.0 mM CaCl_2_, 1.0 μM ZnCl_2_, and 2.5% Triton X-100) and then incubated for 24 h at 37°C in the same buffer at a final concentration of 1%. The gel was then stained with 0.1% Coomassie Brilliant Blue R-250; clear zones against the blue background indicated the presence of gelatinolytic (i.e., collagenase) activity.

### Soft agar assay

Cells were suspended in 0.3% agar medium (DMEM containing 10% FBS) and then plated on a 0.6% agar base layer at a concentration of 1 × 10^3^ cells per six-well plate. The cells were incubated in a humidified atmosphere (5% CO_2_) at 37°C for 10 days, following which the number of colonies that were 50-μm or larger were counted.

### Xenografted tumor model

SW620 cancer cells with stably silenced TPX2 (sh-TPX2) or control (sh-Control and mock) (1 × 10^6^ cells) were subcutaneously injected into the flanks of BALB/c-nu mice as previously described [20]. All procedures involving mice were conducted in accordance with Fudan University Shanghai Cancer Center Animal Care guidelines. All efforts were made to minimize animal suffering, to reduce the number of animals used, and to utilize possible alternatives to *in vivo* techniques.

### Statistics

ANOVA test was used to determine the statistical significance of differences between experimental groups. The Kaplan-Meier method was used to analyze colon cancer patients’ cumulative survival rate. A Cox proportional hazards model was used to calculate univariate and multivariate hazard ratios for the study variables. SPSS software 13.0 (SPSS, Chicago, IL, USA) was used for the analyses. A P value of 0.05 was considered as statistically significant.

## Results

### Aberrant overexpression of TPX2 in colon cancer tissue and cell lines

**Figure 1:**
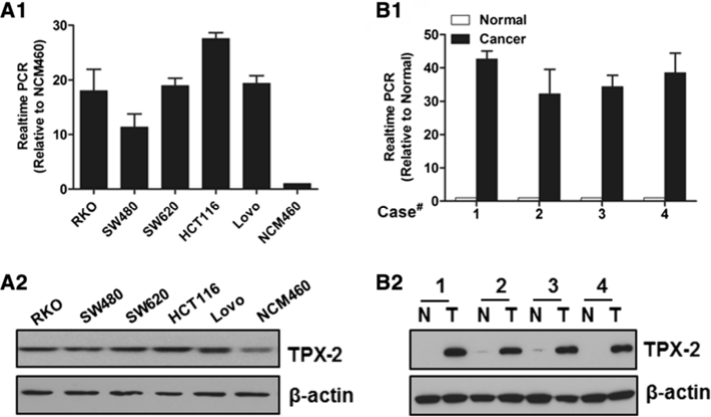
**Analysis of TPX2 expression in colon cancer cell lines and tissues. (A)** Real-time PCR **(A1)** and western blotting **(A2)** analysis were performed to examine TPX2 expression in colon cancer cell lines. **(B)** Real-time PCR **(B1)** and western blotting **(B2)** analysis were performed to examine TPX2 expression in eight cases of primary colon carcinomas (T) and paired non-cancerous adjacent tissues (N).

Real-time PCR analyses revealed that mRNA expression level of TPX2 was markedly higher in all colon cancer cell lines than in non-malignant human NCM460 colonic cell line (Figure 1A1). And the protein expression level of TPX2 was also higher in the colon cancer cell lines but not so markedly as its mRNA expression level (Figure 1A2). Furthermore, comparative analysis showed that the mRNA and protein levels of TPX2 were differentially upregulated in all 4 colon cancer samples compared to the matched adjacent non-tumor tissues (Figure 1B1, B2), suggesting that TPX2 expression is upregulated in colon cancer. The clinicopathologic characteristics of four patients used in western Blot and RT-PCR analysis was provided in the (Additional file 1: Table S1).

### Association between TPX2 expression and the clinical features of colon cancer

**Figure 2:**
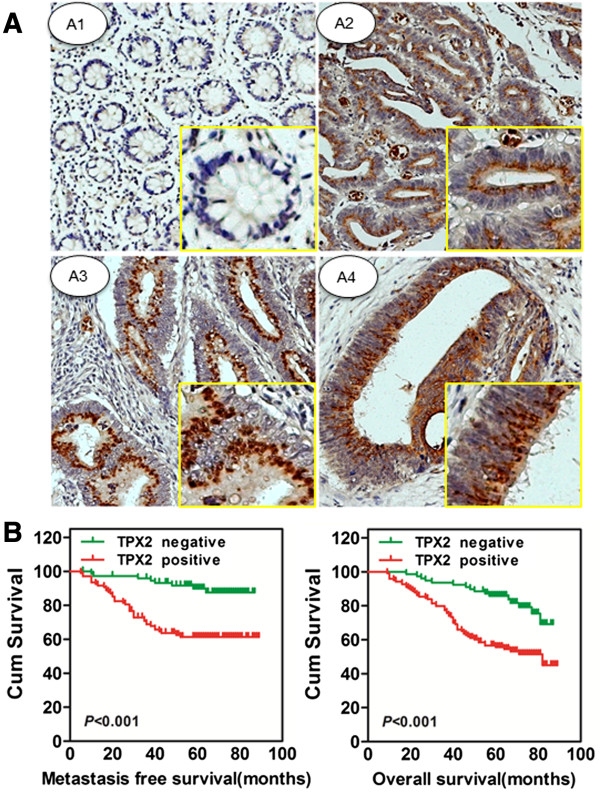
**TPX2 expression and pathologic features in colon cancer specimens. (A)** Immunohistochemical staining of TPX2 in TMA. TPX2 was localized within the nuclei. Elevated TPX2 expression in the tumor cells of colon cancer tissue **(A2, A3,** and **A4)** compared to adjacent normal mucosa with negative staining **(A1)** (original magnification: 400X for the inserts, 100X for all). **(B)** The metastasis-free survival (MFS) and overall survival (OS) rates were estimated by the Kaplan–Meier method. Both the MFS rate and OS rate of patients with TPX2 positive primary tumor were significantly lower than that of patients with TPX2 negative primary tumor (log-rank test, P < 0.001).

To determine whether TPX2 clinically correlated with colon cancer progression, the expression of TPX2 was determined by immunohistochemistry in a tissue microarray containing 203 cases of primary colon cancer paired with their non-cancerous tissue and 66 lymph node metastases (LNM). We found that TPX2 was dramatically upregulated in primary colon cancer (61.1%), but it was either only detected minimally, or not at all (98%) in adjacent normal colonic tissue. The representative expression pattern in both tumor and non-tumor samples are shown in Figure 2A. The quantitative analysis of IHC staining is summarized in Table 1. We observed that the expression levels of TPX2 were closely correlated with the T classification (P = 0.008), lymph node involvement (P = 0.001), distant metastasis (P = 0.048), and clinical stage (P = 0.004) in colon cancer patients. Collectively, these data indicate that TPX2 may be involved in colon cancer carcinogenesis and metastasis.

**Table 1:** Correlation between TPX2 expression and clinicopathologic characteristics

	**Cases**	**TPX2 protein expression(n)**			***P-*** **value**
		**Negative**	**Weak**	**Strong**	
**Age**					
<65	81	33	23	25	0.435
≥65	122	46	28	48	
**Gender**					
Male	86	33	23	30	0.898
Female	117	46	28	43	
**Location**					
Right	84	31	25	28	0.632
Transverse	19	6	6	7	
Left	20	7	6	7	
Sigmoid	80	36	14	31	
**Stage**					
I	24	15	6	3	0.004
II	81	38	20	23	
III	80	23	21	36	
IV	18	3	4	11	
**T stage**					
T1	8	4	2	2	0.008
T2	23	13	6	4	
T3	76	38	17	21	
T4	96	24	26	46	
**N stage**					
N0	108	55	27	26	0.001
N1	61	18	13	30	
N2	34	6	11	17	
**Metastasis**					
M0	185	76	47	62	0.048
M1	18	3	4	11	
**Vessel invasion**					
No	189	74	49	66	0.457
Yes	14	5	2	7	
**Differentiation**					
Well	99	44	26	29	0.108
Moderate	74	27	14	33	
Poor	30	8	11	11	

### TPX2 expression is significantly associated with lymph node metastasis and poor survival in colon cancer patients

Furthermore, we postoperatively analyzed the predictive significance of TPX2 in the development of distant metastasis. The metastasis-free survival (MFS) time was analyzed in 185 patients in stages I-III, who accepted radical colectomy. The proportion of patients who developed metastasis from primary colon cancer after radical colectomy differed substantially between the TPX2-positive and TPX2-negative group (Figure 2B, left). The risk of developing distant metastases after radical colectomy was much higher in patients with a TPX2-positive tumor relative to patients with a TPX2-negative tumor (P < 0.001) (TPX2-positive, 40 [36.7%] of 109 patients, mean follow-up 64.5 months [range 58.4-70.6]; TPX2-negative, 8 [10.5%] of 76, 81.2 [77.3-85.1]). Based on these results, TPX2 could serve as a novel prognostic marker to predict risk of distant metastases in patients with radical colectomy (Hazard Ratio, 4.3; [95% CI 2.0-9.2]; P < 0.001).

A Kaplan-Meier analysis of the data also indicated that the expression of TPX2 was significantly correlated with the overall survival (OS) of colon cancer patients (log-rank test, P < 0.001, Figure 2B, right). Patients with TPX2-positive tumors had a significantly lower 5-year OS than those with TPX2-negative tumors (56.4% vs. 80.6%, HR 3.0 [95% CI 1.7-5.2]. In a multivariate analysis examining clinicopathologic variables such as clinical stage, differentiation grade, and venous invasion, the expression of TPX2 had an independent prognostic value to predict patient outcome, including both the OS and the metastasis-free survival (Table 2).

**Table 2:** Multivariate analysis of metastasis free survival (MFS) and overall survival (OS) of 203 colon cancer patients

**Variable**	**Multivariate analysis (MFS)**			**Multivariate analysis (OS)**		
	***p *** **value**	**HR**	**CI (95%)**	***p *** **value**	**HR**	**CI (95%)**
AJCC stage (I/II vs. III/IV)	<0.001	3.7	1.8-7.5	<0.001	4.6	2.5-8.4
Histologically poorer grade †	0.256	1.6	0.7-3.7	<0.001	3.0	1.7-5.3
Vessel invasion (no vs. yes)	0.022	2.9	1.1-7.4	0.176	1.5	0.8-3.1
TPX2 (negative vs. positive)	0.001	3.7	1.7-8.1	0.006	2.2	1.2-3.9

*N.S.,* Not significant; HR, Hazard ratio; CI, Confidence interval; ^†^ “Histologically poorer grade” corresponded to signet ring cell carcinoma, mucinous adenocarcinoma, and poorly differentiated adenocarcinoma.

### Downregulation of TPX2 inhibits proliferation of colon cancer cells *in vitro* and *in vivo*

**Figure 3:**
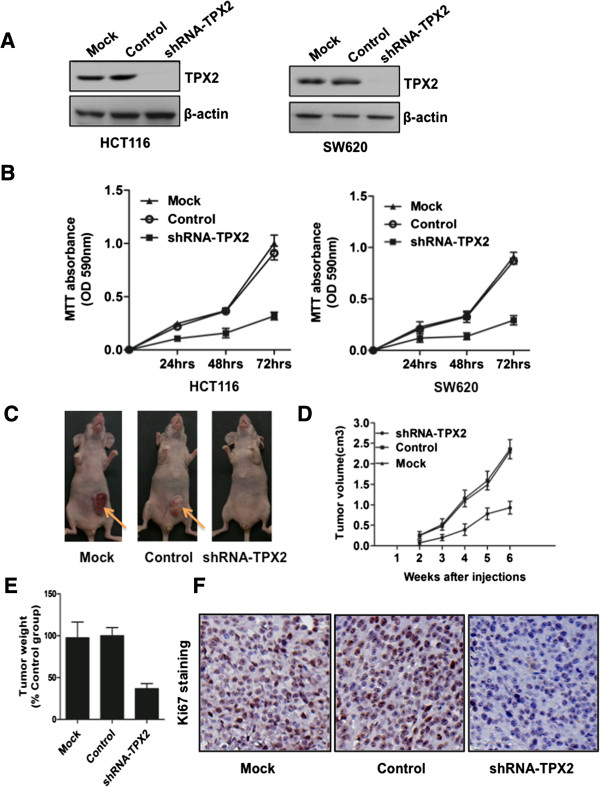
**TPX2 inhibition suppresses proliferation of colon cancer cells *in vitro *and *in vivo. *(A)** HCT116 (left) and SW620 (right) cells were treated with shRNA-TPX2 or control shRNA (control) or Oligofectamine alone (mock) for 48 h, following which cells were harvested for analysis of TPX2 expression by western blot. **(B)** HCT116 (left) and SW620 (right) cells growth curve measured by a 3-(4, 5-dimethylthiazol-2-yl)-2, 5-diphenyltetrazolium bromide (MTT) assay. Each point indicates the mean of the spectrometric absorbance ± S.D. of three independent experiments (P < 0.001). SW620 cells with shRNA-TPX2, shRNA (control) or Mock were injected subcutaneously into the right scapular region of nude mice. Gross tumors in the mice are shown **(C)**; tumors were measured **(D)** and weighted **(E)**, **(F)** Representative Photographs of Ki67 staining in paraffin sections from tumors injected with shRNA-TPX2, or control cells (magnification: 100X).

The impact of TPX2 on proliferation of colon cancer cells was evaluated by knockdown of TPX2. The MTT assay showed that depletion of TPX2 expression caused a marked reduction in the viability of HCT116 and SW620 cells (Figure 3A and B). These results demonstrate that TPX2 suppression could inhibit the proliferation ability of colon cancer cells.

Since TPX2 was correlated with the clinical characteristics of colon cancer, we further investigated the effect of TPX2 on the tumorigenic activity of colon cancer cell lines. Control cells (MOCK and shRNA) and SW620-TPX2-shRNA cells were subcutaneously injected into nude mouse (n = 5/group). As shown in Figure 3C and D, the tumors formed from SW620-TPX2-shRNA cells grew much more slowly than those from the control cells. After 4 weeks, the weight of tumors induced by the TPX2-suppressed cells was significantly reduced when compared to that induced by control cells (Figure 3E). Furthermore, IHC-stained sections of mouse tumors that demonstrated a high level of TPX2 also strongly stained for Ki67 (Figure 3F), consistent with the cell proliferation results *in vitro*. Together, our results indicated that TPX2 plays a critical role in the tumorigenicity of colon cancer cell lines both *in vitro* and *in vivo*.

### Gene Silencing of TPX2 expression in colon cancer cells leads to Akt reduction

**Figure 4:**
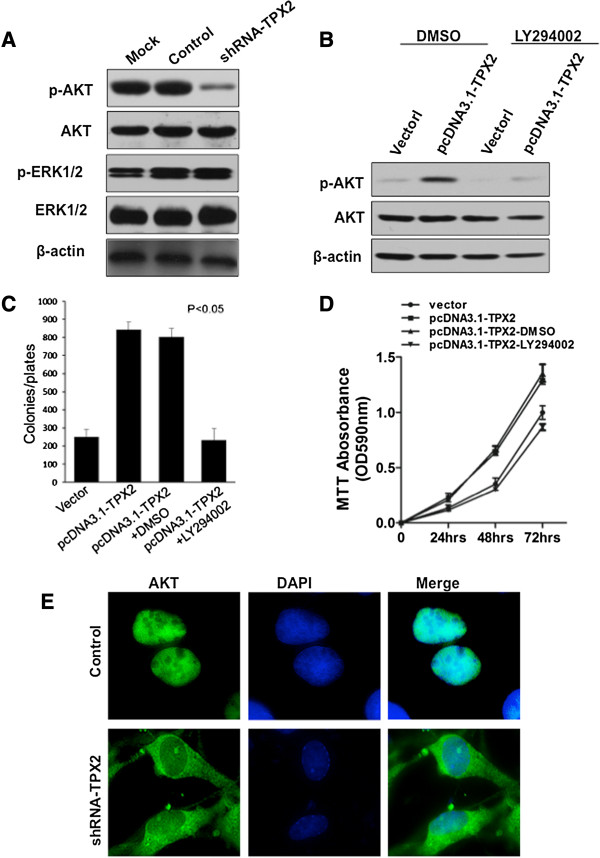
**Inhibition of p-Akt by TPX2 gene silencing in colon cancer cells. (A)** The expression of p-Akt, total Akt, p-ERK-1/2, and ERK-1/2 on SW620 cells treated with shRNA-TPX2, shRNA (control), or Oligofectamine alone (Mock) was analyzed by western blotting. Blocking Akt activation decreases the colony formation induced by TPX2. SW480 cells were transfected with pcDNA3.1 or pcDNA3.1-TPX2 and then treated with LY294002 (25 μmol/L) for 2 h. **(B)** p-Akt and total Akt was analyzed by western blotting. **(C)** The colony formation ability of the cells was determined by a colony formation assay. The bars indicate mean colony numbers from a representative experiment done in triplicate; with SD. P < 0.05 compared to the no-treatment group. **(D)** Cell growth curve was measured by a 3-(4, 5-dimethylthiazol-2-yl)-2, 5-diphenyltetrazolium bromide (MTT) assay. Each point indicates the mean of the spectrometric absorbance ± S.D. of three independent experiments (P < 0.01) **(E)** Immunofluorescence microscopic analyses of Akt activation. SW620 cells were infected with shRNA-TPX2 or control cells (control shRNA and mock). Then, the cells were immunostained for Akt (green) and their nuclei were stained with 4′,6-diamidino-2-phenylindole (DAPI; blue).

As TPX2 expression is linked to poor survival of colon cancer patients, we wanted to further explore the molecular mechanism of its action. We found that the phosphorylation and activation of Akt was markedly reduced in shRNA-TPX2 transfected cells compared with the control group, while downregulation of TPX2 did not affect ERK-1/2 activation, which are involved in a different pathway from Akt (Figure 4A). Furthermore, knocking down TPX2 in SW620 reduced nuclear Akt (Figure 4E).

To confirm whether TPX2 induced proliferation of colon cancer cells through the Akt pathway, we overexpressed TPX2 in SW480, which is a lower grade colon cancer cell line, then treated with a phosphoinositide 3-kinase (PI3K) inhibitor LY294002. Blockade of Akt activation suppressed the proliferation induced by TPX2 in SW480 cells, as determined by a colony formation assay and MTT assay (Figure 4B, C and D). Together, these data suggest that downregulation of TPX2 inhibits Akt activation, and Akt activation is an important step in the TPX2-induced proliferation of colon cancer cells.

### Gene silencing of TPX2 suppresses the migratory and invasive ability of colon cancer cells through a modulation of MMP2 expression and activity

**Figure 5:**
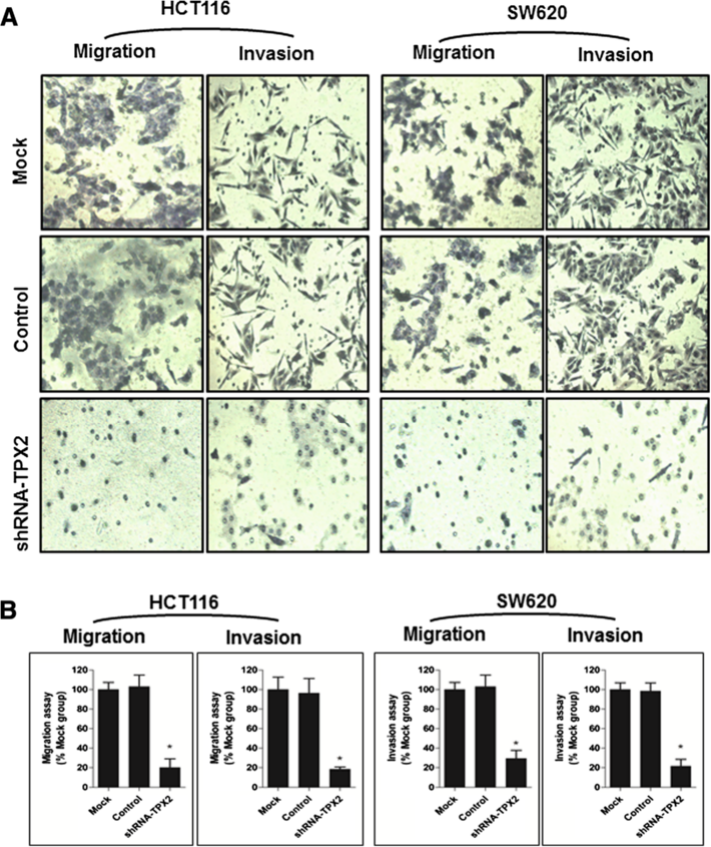
**TPX2 inhibition suppressed invasion and migration of colon cancer cells. (A)** Representative images show migration and invasion of HCT116 and SW620 cells infected with shRNA-TPX2, shRNA (control), or Oligofectamine alone (mock) in a transwell assay (200×). **(B)** Data are presented as mean relative numbers of migrated cells from 4 fields (*P < 0.01, versus mock group).

As TPX2 is linked to the advanced clinical stage and poorer MFS of colon cancer patients, we then wanted to determine the possible role of TPX2 on cell migration and invasion activity *in vitro*. The effect of TPX2 knockdown on migration potency of SW620 cells was assayed using migration chambers. Compared to the control groups, TPX2 silencing resulted in significantly reduced migratory ability (P <0.01, Figure 5A and B). We also assessed the effect of TPX2 depletion on tumor invasion and demonstrated that disruption of endogenous TPX2 expression also attenuated cell invasive potential in colon cancer cells (Figure 5A and B). The results indicate a critical role of TPX2 in the metastasis of colon cancer.

**Figure 6:**
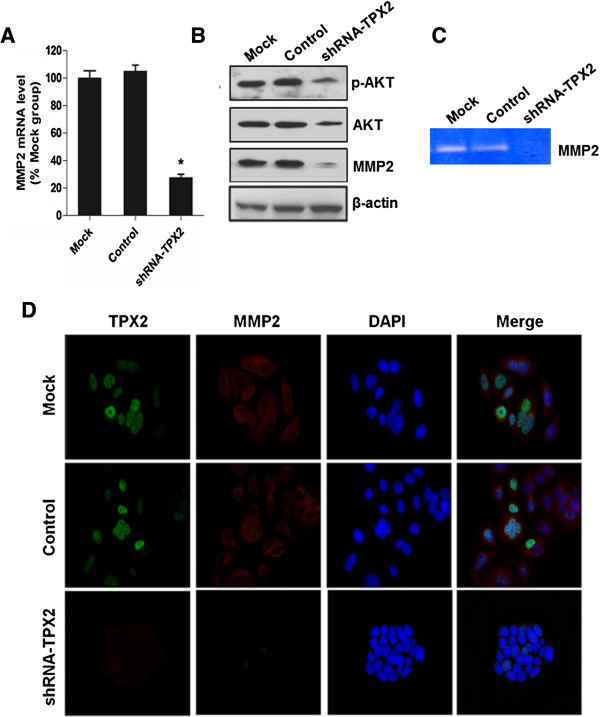
**Gene silencing of TPX2 expression in colon cancer cells suppresses MMP-2 activity.** The expression or enzyme activity of TPX2 on SW620 cells treated with shRNA-TPX2, shRNA (control), or Oligofectamine alone (mock) was analyzed by RT-PCR **(A)**, western blot **(B)**, and zymography assay **(C)**. **(D)** Immunofluorescence microscopic analyses of MMP-2. SW620 cells were infected with shRNA-TPX2 or control cells (control shRNA and mock). The cells were then immunostained for TPX2 (green) and MMP-2 (red), their nuclei were stained with 4′,6-diamidino-2-phenylindole (DAPI; blue).

To better understand the role of TPX2 in the progression and metastasis of colon cancer cells, we explored the possible roles of metastasis-related molecules downstream of TPX2. We found that knockdown of endogenous TPX2 led to substantial reduction in both mRNA and protein level of MMP2 (Figure 6A, B, and D). We next examined the potential effect of TPX2 on the activity of MMP2 using zymography analysis. Higher activity of MMP2 was observed in control group compared to ShRNA-TPX2 treated cells (Figure 6C). The data suggest that TXP2 may be a potential target in colon cancer therapy due to its ability to modulate downstream MMP2 expression and activity.

## Discussion

The motor-binding targeting protein for Xklp2 (TPX2) is the first cell cycle- associated protein with a restricted pattern of expression and high level of activity found in several malignant tumors [21,22]. Aberrant expression of TPX2 has been associated with both malignant transformation of respiratory epithelium and progression of squamous cell lung cancer [23,24]. It has been shown that the TPX2 gene is amplified in pancreatic tumor tissues and may serve as biomarker for identifying subpopulations of patients sensitive to Aurora-A inhibitor treatment in Non-Hodgkin’s lymphoma [25,26]. However, little work has been done to explore the role of TPX2 in colon cancer.

This study has shown for the first time that aberrant expression of TPX2 is significantly associated with unfavorable clinicopathologic variables of colon cancer and that overexpression of TPX2 leads to the activation of Akt, a mechanism by which TPX2 promotes proliferation and tumorigenesis. The study also shows that TPX2 plays a critical role in the progression and metastasis of colon cancer, which could be mechanistically associated with activity of MMP2 and finally, that TPX2 protein expression could serve as a novel biomarker to predict the risk of metastasis in colon carcinoma patients after a colectomy.

Tumorigenesis, characterized by uncontrolled cell growth and tumor formation is associated with alterations in genes or proteins related to regulation of proliferation, cell death, and genomic stability [27,28]. Thus, identification of genes and their products involved in the molecular events leading to tumorigenesis is critical to developing effective therapeutic strategies. In our study, we found that TPX2 was a potential marker involved in tumorgenesis of colon cancer. TPX2 was markedly upregulated in colon cancer cells and tissues. Moreover, silencing of TPX2 reduced the tumorigenicity of colon cancer cells both *in vitro* and *in vivo*, implicating TPX2 as an oncogenic protein in the development and progression of colon cancer. Here we report further that decreased expression of TPX2 in colon cancer cell line SW620 caused a significant decrease in the level of p-Akt, which is an important signaling pathway for tumor formation. Moreover, the PI-3 K-specific inhibitors LY294002 can inhibit TPX2-induced colony formation *in vitro*. Therefore, TPX2 may cause proliferation of colon cancer cells through an activation of the PI3K/Akt signaling pathway, a potential therapeutic target.

In addition to playing a critical role in cancer cell proliferation and tumorigenesis, TPX2 appears to be involved in metastasis, as it is tightly cell-cycle regulated [29,30]. Our study observed that TPX2 expression was closely associated with tumor stage and lymph node metastasis in colon cancer, suggesting that TPX2 could be important in colon cancer progression. Invasion and metastasis are characteristic features of colon cancer and the main factors related to the poor prognosis in patients with colon cancer [31]. Thus, the identification of the molecular mechanisms responsible for the control of the invasive and metastatic potential of colon cancer is crucial to inhibit these processes. In the present study, we explored whether TPX2 contributed to migration and invasion of colon cancer cells *in vitro*. Our data revealed that depletion of TPX2 could suppress colon cancer cell migration and invasion in vitro. These results suggest that TPX2 plays an important role in invasion and metastasis of colon cancer and that TPX2 may be a new and crucial therapeutic target for colon cancer.

The degradation of ECM is a critical step in tumor invasion and metastasis. Matrix metalloproteases (MMPs), a family of zinc-dependent endopeptidases, play a major role in the degradation of ECM components [32,33]. Among these MMPs, matrix metalloproteinase-2 (MMP2) has been considered critical for cancer invasion and metastasis [34,35]. Here we found that downregulation of TPX2 could diminish the expression of MMP2, both at the mRNA and protein levels. It has been reported that the phosphatidylinositol 3-kinase/Akt signaling pathway plays a critical role in promoting MMP-2 expression [36,37]. Therefore, these results suggest that the downregulation of TPX2 could potentially inhibit the tumorigenesis and metastasis of colon cancer, partially through PI3K/Akt pathway and MMP-2.

## Conclusion

In summary, we show here for the first time that TPX2 is highly expressed in colon cancer tissues and cell lines. Inhibition of TPX2 expression inactivates the PI3K/Akt signaling pathway and reduces tumorigenicity of colon cancer cells. It also results in the downregulation of MMP2, resulting in reduced metastasis. These results suggest that TPX2 expression is critical for the progression and invasiveness of colon cancer. Since TPX2 has multiple roles in the progression of colon cancer, including regulation of proliferation, invasion, and metastasis of colon cancer cells, the frequent upregulation of TPX2 in human colon cancers highlights its importance as a novel therapeutic target in the treatment of colon cancer.

## Competing interests

The authors declare that they have no competing interests.

## Authors’ contributions

All authors participated in the design, interpretation of the studies and analysis of the data and review of the manuscript; DL, SC, PW and NZ conducted the experiments, YX, XL supplied critical reagents for immunohistochemistry, DS, YW conducted *in vitro* experiments, PW wrote the manuscript. All authors read and approved the final manuscript.

## Supplementary Material

Additional file 1: Table S1. Clinicopathologic characteristics of four patients used in western Blot and RT-PCR analysis. Click here for file
