# Supplementary material for: TPX2 is a novel prognostic marker for the growth and metastasis of colon cancer
Source: J Transl Med. 2013 Dec 17;11:313. doi: 10.1186/1479-5876-11-313 (PMC3878622; doi:10.1186/1479-5876-11-313)
Supplement: Additional file 1: Table S1. — Clinicopathologic characteristics of four patients used in western Blot and RT-PCR analysis. [file 1479-5876-11-313-S1.doc]

Additional file 1 Table S1 Clinicopathologic characteristics of four patients used in western Blot and RT-PCR analysis

|  | *Case1* | *Case2* | *Case3* | *Case4* |
| --- | --- | --- | --- | --- |
| Age | 45 | 67 | 63 | 58 |
| Gender | Female | Male | Male | Female |
| Location | Right | Right | Transverse | Sigmoid |
| Stage | Ⅱ | Ⅱ | Ⅲ | Ⅲ |
| pTNM | T3N0M0 | T3N0M0 | T3N1M0 | T4N1M0 |
| Vessel invasion | No | No | No | Yes |
| Differentiation | Moderate | Well | Moderate | Poor |
